# Antiphospholipid Syndrome Coexisting With Evans Syndrome and SCL‐70 Antibody Positivity: A Case Report

**DOI:** 10.1002/ccr3.71723

**Published:** 2025-12-29

**Authors:** Chunhua Cao, Xiaodong Hu, Zian Li, Deqing Li, Jideng Ma

**Affiliations:** ^1^ Department of Clinical Laboratory Qinghai Provincial Peoples Hospital Xining China; ^2^ Qinghai Provincial Police College Xining China

**Keywords:** antiphospholipid syndrome, Evans syndrome, SCL‐70 antibody, systemic sclerosis

## Abstract

This case of antiphospholipid syndrome, Evans syndrome, and anti‐Scl‐70 positivity (suggesting early SSc) showed marked symptom improvement with rituximab, but poor prognosis persists. Highlights the need for early diagnosis of overlapping autoimmunity, SSc complication monitoring, and optimized immunotherapy in complex cases.

## 1 Introduction

Antiphospholipid syndrome (APS) is an autoimmune disorder characterized by the presence of antiphospholipid antibodies (aPL), typically occurring concurrently with or secondary to other autoimmune diseases such as systemic lupus erythematosus (SLE) or Sjögren's syndrome. However, in some cases, APS may present as an isolated primary condition without concomitant autoimmune diseases. This form is termed primary APS, exhibiting diverse clinical manifestations including thrombosis, pregnancy complications, and hematological abnormalities [1, 2].

The incidence of autoimmune cytopenias (including autoimmune hemolytic anemia, AIHA, and immune thrombocytopenia, ITP) remains relatively low among APS patients [3]. According to existing research, the prevalence of AIHA in APS patients ranges from approximately 6% to 21%, while Fisher–Evans syndrome (the coexistence of AIHA and ITP) occurs in about 10%–15% of cases [4]. Fisher–Evans syndrome is a rare yet severe hematological complication characterized by the simultaneous presence of AIHA and ITP, which may manifest as one of the clinical presentations in APS patients [5].

The relationship between APS and Evans syndrome (ES) has garnered increasing attention in recent research. The presence of aPL in APS patients may induce hematological abnormalities, including thrombocytopenia and hemolytic anemia. These antibodies bind to phospholipids on the surfaces of red blood cells and platelets, activating the complement system and leading to cellular destruction. This mechanism plays a pivotal role in the development of both AIHA and ITP [3].

Positive anti‐scleroderma‐70 antibody (SCL‐70) status is typically associated with systemic sclerosis (SSc) and constitutes one of its hallmark antibodies [6]. Several studies have demonstrated a significantly higher prevalence of SCL‐70 positivity in APS patients compared to non‐APS individuals, suggesting a potential association between APS and SSc. Specifically, one study found an SCL‐70 positivity rate as high as 71% in APS patients, markedly exceeding that in non‐APS patients (34%). This suggests SCL‐70 positivity in APS may correlate with disease progression and clinical manifestations [7].

This case report aims to explore the clinical presentation and management of APS presenting as an initial, independent symptom followed by secondary development of Fisher–Evans syndrome through a specific clinical case. By detailing the clinical features, laboratory findings, and treatment course of this case, we aim to enhance clinicians' awareness of APS and its rare complications, emphasizing the importance of early diagnosis and intervention. Furthermore, we discuss potential mechanisms linking APS and Fisher–Evans syndrome, alongside strategies for identifying and managing such complex presentations in clinical practice.

## 2 Case History/Examination

### 2.1 Case History

A 37‐year‐old female patient was admitted for 9 days of irregular vaginal bleeding. She had previously experienced regular menstrual cycles, but over the last 2 months, her periods had lengthened, and the flow volume had increased. There were no associated symptoms such as dizziness or abdominal pain. On physical examination, the patient presented with signs of anemia, including pale skin and mucous membranes, fatigue, increased heart rate, and shortness of breath after physical activity, dizziness, blurred vision, lack of concentration, reduced appetite, and multiple scattered petechiae and ecchymoses over the body. The patient reported that over the past 6 months, she had experienced recurrent Raynaud's phenomenon (cyanosis and redness of the hands) upon contact with cold water, occasionally accompanied by swelling. The patient had a history of hypertension for 7 years, with blood pressure reaching up to 180/100 mmHg. She was diagnosed with cerebral infarction 3 years ago. Four months prior, she suffered multiple cerebral infarctions accompanied by seizures. CT findings at that time revealed multiple infarct lesions in the right frontal–parietal lobe and periventricular regions of both lateral ventricles, a softening lesion in the right frontal lobe, demyelinating changes in the cerebral white matter, and cerebral atrophy. Preliminary diagnoses included thrombocytopenia, severe anemia, abnormal uterine bleeding, cerebral atrophy, cerebral infarction, and epilepsy. The family history was unremarkable, with her parents being in good health and having no similar medical history.

### 2.2 Examination

**FIGURE 1:**
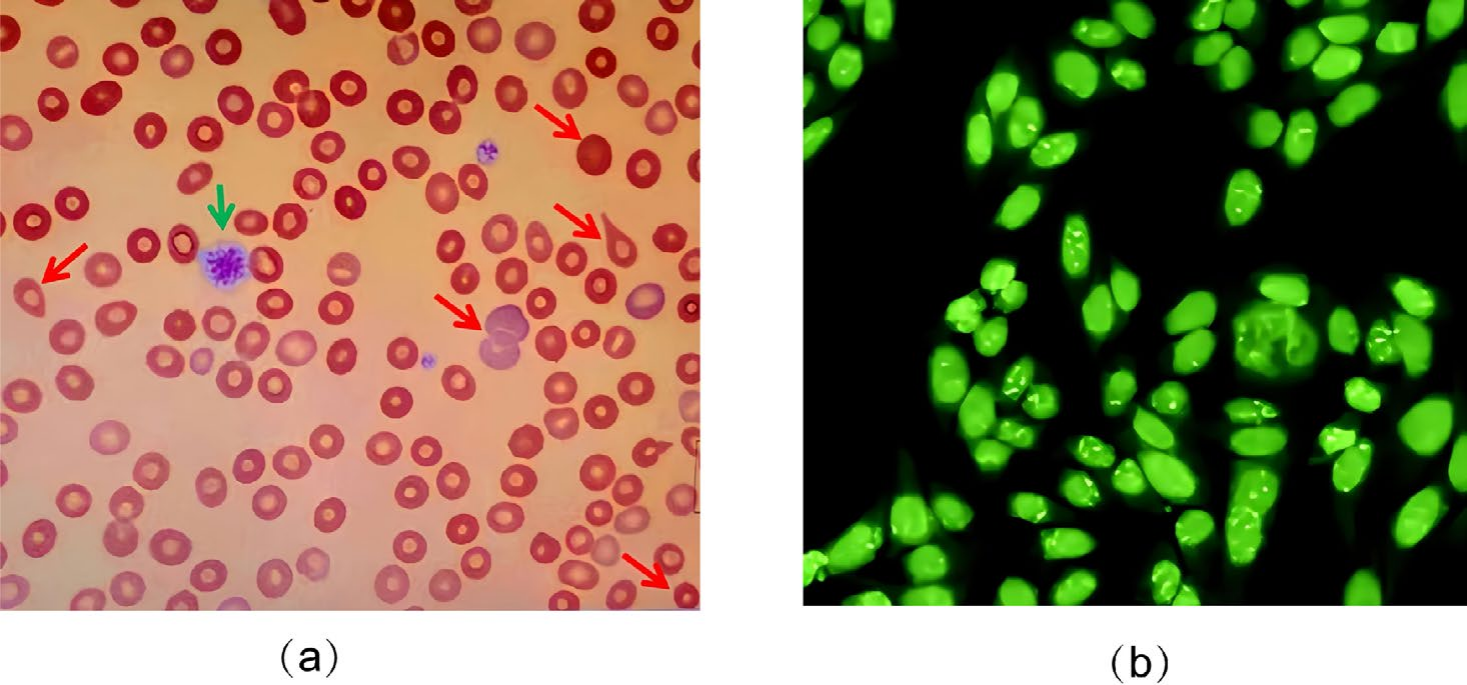
Hematological features, and immunological findings in this case. (a) Microscopic examination of peripheral blood revealed erythrocytes of uneven size and various abnormal forms (10 × 10). (b) ANA test was positive, showing TOP 1 type at a titer of 1:1000.

The routine blood test showed erythrocytes at 1.69 × 10^12^/L, hemoglobin at 55 g/L, platelets at 2 × 10^9^/L, and reticulocytes increased to 156 × 10^9^/L. Peripheral blood smear microscopy revealed erythrocytes of varying sizes, with a range of abnormal forms, including a small number of teardrop‐shaped erythrocytes, pleochroic erythrocytes, and spherocytes (Figure 1a); mean corpuscular volume (MCV) 96.5 fL; mean corpuscular hemoglobin concentration (MCHC) 32.6 pg; white blood cell count (WBC) 4.57 × 10^9^/L; absolute neutrophil count 3.79 × 10^9^/L; neutrophil percentage; 83.10%; prothrombin time (PT) 12.7 s; Activated partial thromboplastin time (APTT) 21.8 s; HCG test negative; The diagnosis AIHA was confirmed by elevated GGT, ALP, lactate dehydrogenase (LDH) levels, and a positive Coombs test. Platelet‐specific antibody test results were positive. Bone marrow cytomorphology revealed active nucleated cell hyperplasia, characterized by a marked proliferation of the granulocyte and erythrocyte lineages. Megakaryocytosis with impaired maturation was observed. Given the presence of two autoimmune cytopenias (hemolytic anemia and thrombocytopenia), a preliminary diagnosis of ES was made (Table 1).

**TABLE 1:** Laboratory results in the case report.

Tests	Results	RI	Tests	Results	RI
ACL‐IgA	1.09 U/mL	< 12	cAIHA	Negative	Negative
ACL‐IgG	0.66 U/mL	< 12	dsDNA	Negative	Negative
ACL‐IgM	2.59 U/mL	< 12	GGT	105.7 U/L	7–45
ANA: AC‐1	1:1000	Negative	HB	55 g/L	119–157
ANA: Scl‐70	229.50 RU/ml	0–20	HBDH	238 U/L	72–182
ANC	3.79 × 10^9^/L	1.8–6.3	HCG	3 IU/L	0–5
APTT	21.8 s	23.3–32.5	HCT	16.3%	38.0–49.0
β2‐GPIIgA	61.04 RU/ml	< 20	HLA	Positive	Negative
β2‐GPIIgG	87.79 RU/ml	< 20	IL‐6	32.9 pg/mL↑	0–7
β2‐GPIIgM	6.32 RU/ml	< 20	LA1	53.3 (sec)↑	31–44
CD3	20.21↓	56–86	LA2	37.7 (sec)	0.8–1.2
CD3 + CD8+	11.44↓	13–39	LA	Positive	Negative
CD3 + CD4+	8.40↓	33–58	LDH	300 U/L	120–250
Coombs	Anti‐IgG (2+)	Negative	NEUT%	83.10%	40–75
	Anti‐IgG, C3d (3+)	Negative	PLT	2 × 109/L	70–300
	Anti‐C3d (2+)	Negative	PT	12.7 s	10–14
CD19+	70.99↑	5–26	PCT	0.074 ng/mL↑	0.0–0.046
CD16 + CD56+	4.34↓	5–22	RBC	1.69 × 10^12^/L	3.90–5.30
C3	62 mg/dI↓	90–180	RET	156 × 10^9^/L	24–84
C4	8 mg/dI↓	Negative	WBC	4.57 × 10^9^/L	3.50–9.50

Abbreviations: ACL‐IgA, anticardiolipin antibodyL‐immunoglobulin A; ACL‐IgG, anticardiolipin antibodyL‐immunoglobulin G; ACL‐IgM, anticardiolipin antibodyL‐immunoglobulin M; ANA, antinuclear antibodies; ANC, absolute neutrophil count; APTT, activated partial thromboplastin time; cAIHA, cold autoimmune hemolytic anemia; dsDNA, double‐stranded DNA; g/L, grams per liter; GGT, gamma‐glutamyltransferase; HB, hemoglobin; HBDH, α‐hydroxybutyrate dehydrogenase; HCG, human chorionic gonadotropin; HCT, hematocrit; HLA, platelet‐specific and tissue‐relevant affinity antibodies; IL‐6, interleukin‐6; L, liter; LA, lupus anticoagulant; LDH, lactic dehydrogenase; mg/dl, milligrams per deciliter; NEUT%, neutrophil percentage; ng/dl, nanograms per deciliter; PCT, procalcitonin; pg/dl, picograms per deciliter; PLT, platelet; PT, prothrombin time; RBC, red blood cells; RET, reticulocyte; RU/ml, reference unit per milliliter; U, units; U/L, unit per liter; U/ml, Unit per milliliter; WBC, white blood cells; β2‐GPI IgA, beta 2 glycoprotein I immunoglobulin A; β2‐GPIIgG, beta 2 glycoprotein I immunoglobulin G; β2‐GPIIgM, beta 2 glycoprotein I immunoglobulin M.

Given the patient's background as a young woman with recurrent cerebral infarctions and treatment‐resistant thrombocytopenia, the laboratory assessment confirmed several key findings: a positive ANA test exhibiting a speckled pattern associated with the TOP 1 antigen at a significant titer of 1:1000 (Figure 1b). The ENA antibody profile was negative for all antibodies except Scl‐70, which was positive. Anti‐dsDNA antibody was negative, lupus anticoagulant was positive, anti‐β2‐glycoprotein antibody was positive at high titer, and anticardiolipin antibody was within normal range. These results, in conjunction with her clinical history, fulfilled the criteria for the diagnosis of APS. Further immune profiling through lymphocyte subset analysis revealed impaired T‐cell function, enhanced B‐cell activity, and a CD4+/CD8+ ratio below the normal threshold of 1.0, indicative of profound immune dysregulation. Serum complement levels (C3 and C4) were reduced, indicating active disease. SCL‐70 test results were positive, and chest CT findings showed bilateral pleural effusions (likely due to the patient's chronic anemia) with no evidence of pulmonary fibrosis.

## 3 Differential Diagnosis and Treatment

### 3.1 Differential Diagnosis

The patient presented with manifestations primarily including anemia and thrombocytopenia, without significant leukocyte abnormalities, and was found to have markedly active bone marrow hyperplasia. This clinical picture necessitated a systematic evaluation to distinguish between several potential hematologic and systemic disorders.

Aplastic anemia (AA) is characterized by a bone marrow failure syndrome, resulting in pancytopenia and manifestations of anemia, hemorrhage, and infections [8]. The hallmark diagnostic feature is bone marrow hypocellularity [9, 10]. However, this case was not compatible with a typical AA presentation due to the observed markedly active bone marrow hyperplasia, absence of pancytopenia (as leukocyte counts were within normal limits), and the lack of severe hemorrhagic or infectious complications at presentation.

SLE is a systemic autoimmune disorder capable of inducing hematologic abnormalities such as cytopenias (e.g., leukopenia, anemia, thrombocytopenia) [10, 11]. The patient's ANA pattern was atypical (Topo‐I type), and specific autoantibodies highly characteristic of SLE—such as anti‐dsDNA and anti‐Sm—were absent. Furthermore, there were no clinical signs suggestive of SLE, such as skin rashes, photosensitivity, oral ulcers, or involvement of other organ systems like the kidneys or joints.

MDS comprises a group of heterogeneous clonal hematopoietic stem cell disorders [12]. Its diagnosis fundamentally relies on the presence of morphological dysplasia in one or more myeloid cell lineages, ineffective hematopoiesis leading to peripheral cytopenias, and a risk of progression to acute myeloid leukemia (AML) [13]. In this patient, careful morphological examination of peripheral blood and bone marrow samples did not reveal significant dysplastic features in any of the myeloid lineages (erythroid, granulocytic, and megakaryocytic).

### 3.2 Investigations and Treatment

The patient was treated with methylprednisolone, intravenous immunoglobulin, recombinant human thrombopoietin, component blood transfusion, and hemostatic drugs. Vaginal bleeding stopped after diagnostic curettage, but hemoglobin and platelet counts showed little improvement.

**FIGURE 2:**
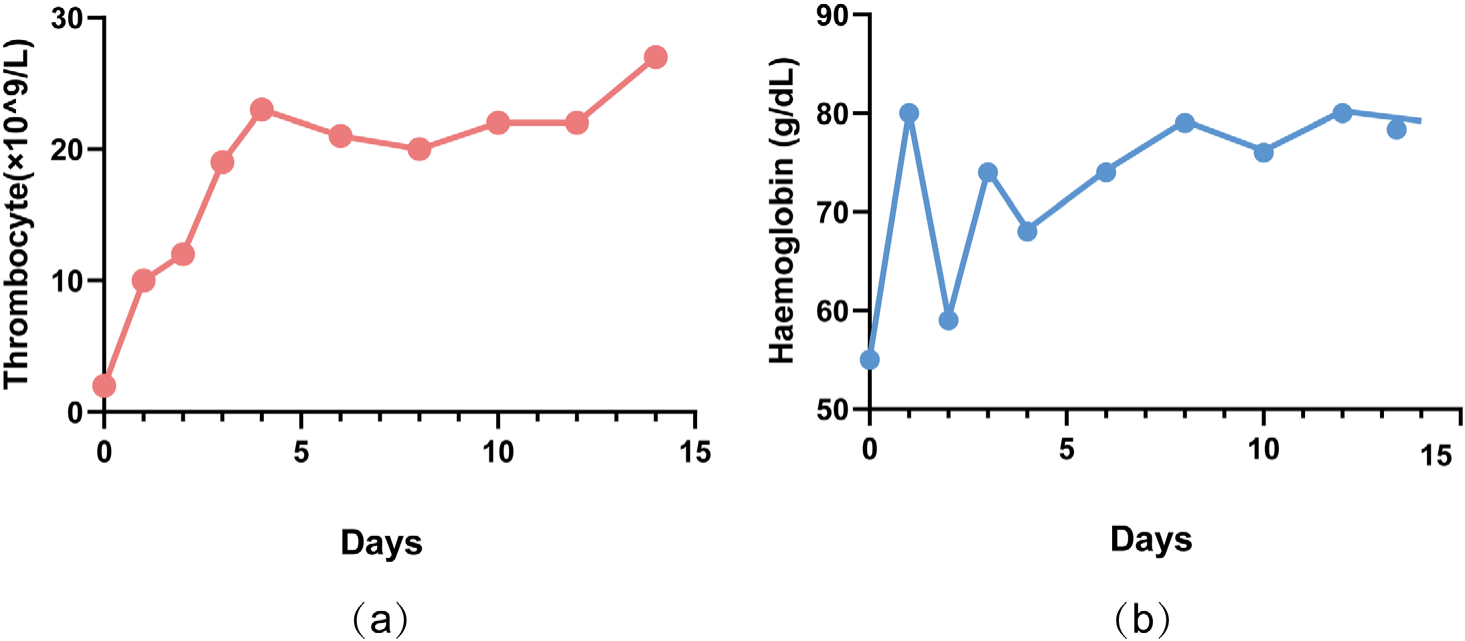
Thrombocytes and hemoglobin levels during hospitalization. (a) This graph illustrates the changes in thrombocyte count during the hospitalization period. (b) This graph depicts the changes in hemoglobin level during hospitalization.

Subsequent treatment included intravenous immunoglobulin pulse therapy and rituximab to inhibit B‐cell activity. After 2 weeks, hemoglobin and platelet levels increased significantly, bleeding symptoms improved markedly (Figure 2), long‐term oral vitamin K antagonists (such as warfarin, with an INR target range of 2.0–3.0), or low molecular weight heparin combined with aspirin, and the patient's family requested discharge.

## 4 Outcome and Follow‐Up

### 4.1 Outcome

The combination of these findings confirmed the diagnosis of APS combined with ES, and early SSc was considered.

### 4.2 Follow‐Up

**FIGURE 3:**
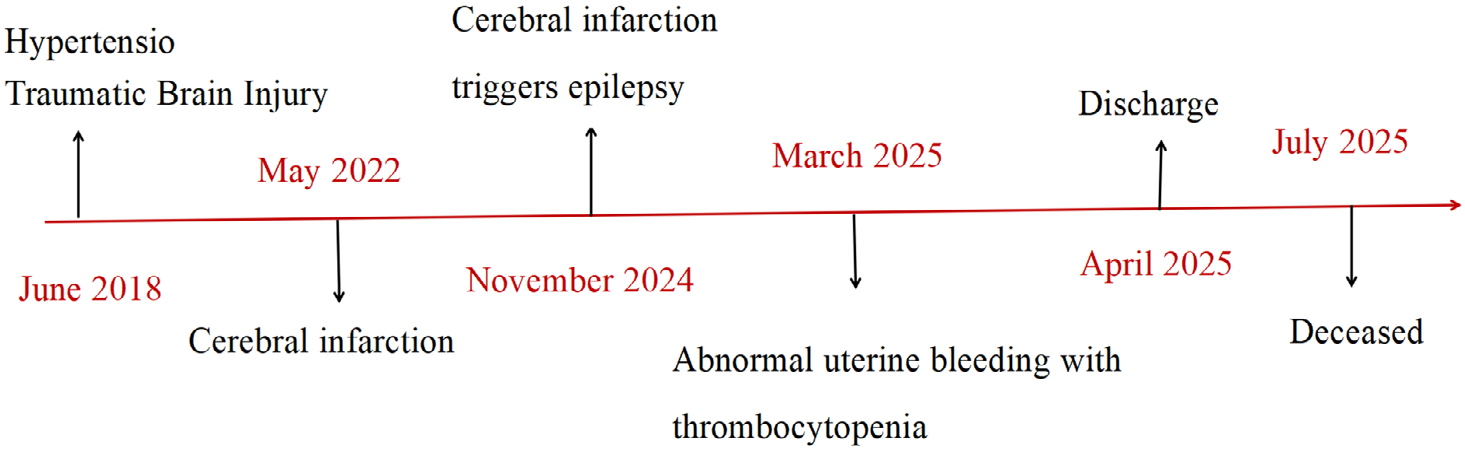
Timeline of key events in the case history.

The patient was instructed to review regularly, including blood routine and reticulocytes, liver and kidney function, electrolytes, and autoantibodies, and to have regular follow‐up for recurrence of hemolytic crisis or thrombosis. The patient passed away 3 months after discharge. Figure 3 presents a detailed timeline of the key events that occurred.

## 5 Discuss

ES is rare, and its combination with APS and SSc is even more infrequent. ES can present as primary or secondary to other disorders, commonly secondary to lymphoma, autoimmune diseases (e.g., SLE, rheumatoid arthritis, and APS), or infections (hepatitis viruses, Epstein–Barr virus (EBV), cytomegalovirus, and human immunodeficiency virus), among others. Comprehensive superficial lymph node examination revealed no abnormalities, and serological tests for EBV, hepatitis viruses, syphilis, and human immunodeficiency virus were negative, excluding associations with lymphoma and viral infections. These findings supported a diagnosis of ES secondary to APS.

The patient exhibited high‐titer positivity for SCL‐70 antibody, a key serological marker in diffuse SSc with a reported prevalence of 9.4%–42%. This antibody is often associated with poor prognosis and may precede clinical symptoms by months to years. Although the patient lacked obvious cutaneous sclerosis and lung CT showed no evidence of fibrosis, the SCL‐70 positivity suggested a potential early stage of SSc.

APS and ES are both autoimmune disorders with overlapping pathogenic mechanisms and clinical features, which can be summarized as follows: (1) Autoantibody cross‐reactivity, APS is characterized by aPL, which promote thrombosis and pregnancy complications [14]. Research indicates that some ES patients harbor aPL, suggesting shared immune pathways for autoantibody production between the two syndromes [15]. (2) Dysregulated immune activation, both disorders stem from immune system dysfunction, potentially triggered by genetic, infectious, or environmental factors. This leads to aberrant immune responses against self‐cells, such as B‐cell hyperactivity or T‐cell regulatory defects [16, 17]. (3) Overlapping clinical manifestations, ES presents with core features of hemolytic anemia and thrombocytopenia (bleeding tendency). APS patients may exhibit thrombocytopenia or even concomitant hemolytic anemia in addition to thrombotic risk [18], overlapping with the hematological symptoms of ES. These disorders may exist independently or comorbidly due to shared immune mechanisms [19].

Cases of ES combined with antiphospholipid antibody syndrome (APS) are rare. One was a life‐threatening case of ES due to antiphospholipid antibody syndrome [20], and the other was ES coexisting with SLE and APS [21]. The present case was the first reported case of APS combined with ES, characterized by positive SCL‐70 antibodies. The case was successfully treated with a multimodal treatment regimen based on rituximab. These cases are potentially fatal and often require high‐dose combination immunosuppressive therapy. Glucocorticoids (GC) and intravenous immunoglobulin (IVIG) were the first‐line regimen, and second‐line treatment included immunosuppressive agents and rituximab, a monoclonal antibody [22]. Rituximab‐based regimens offered curative possibilities and were safe for use in severe APS with ES in patients who did not respond well to conventional therapy, confirming the key role of B‐cell‐mediated autoimmunity in the pathogenesis of APS [16].

Beyond anticoagulants and immunosuppressants, recent research has highlighted other potential therapeutic targets within the innate immune system, including the complement system and immunomodulatory therapies [23]. Studies indicate that the complement system plays a significant role in the pathogenesis of APS, with complement inhibition potentially exerting therapeutic effects on cytopenias through mechanisms such as suppressing inflammatory responses, regulating immune complex deposition, protecting hematopoietic stem and progenitor cells, and improving vascular endothelial function [24].

This case presents significant educational value, though certain limitations exist: (1) The absence of long‐term data precludes assessment of whether SCL‐70 positivity progresses to overt SSc (e.g., pulmonary fibrosis or renal crisis) and hinders evaluation of the long‐term efficacy of immunosuppressive therapy. (2) Although ES was confirmed via bone marrow aspiration, the absence of capillary microscopy and skin biopsy precluded clarification of the specific pathological features of vascular lesions or cutaneous sclerosis. (3) Testing was limited to classical aPL (e.g., lupus anticoagulant, anticardiolipin antibodies), excluding novel antibodies (e.g., anti‐membranin A5 antibody).

This case presents a clinical model of the rare combination of APS with ES and positive anti‐SCL‐70 antibodies, highlighting the significance of overlapping immune mechanisms in complex autoimmune disorders. Future research should focus on optimizing long‐term follow‐up strategies, expanding antibody detection panels, and exploring the value of targeted therapies such as complement inhibitors.

## Author Contributions

**Chunhua Cao:** conceptualization, data curation, investigation, writing – original draft. **Zian Li:** funding acquisition, resources, supervision. **Deqing Li:** conceptualization, investigation. **Jideng Ma:** conceptualization, data curation, supervision, writing – original draft, writing – review and editing.

## Funding

This work was supported by National Clinical Key Specialty in Medical Laboratory Science, and National Clinical Key Specialty Project in Medical Laboratory of the People's Republic of China (QWSJB‐2024‐90).

## Consent

Written informed consent for publication of this case report was obtained from the patient. Consent was documented on a Chinese consent form rather than on the journal's template.

## Conflicts of Interest

The authors declare no conflicts of interest.

## Data Availability

The data that supports the findings of this study are available on request from the corresponding author. The data are not publicly available due to privacy or ethical restrictions.
